# [^99m^Tc]Tc-DB1 Mimics with Different-Length PEG Spacers: Preclinical Comparison in GRPR-Positive Models

**DOI:** 10.3390/molecules25153418

**Published:** 2020-07-28

**Authors:** Panagiotis Kanellopoulos, Emmanouil Lymperis, Aikaterini Kaloudi, Marion de Jong, Eric P. Krenning, Berthold A. Nock, Theodosia Maina

**Affiliations:** 1 Molecular Radiopharmacy, INRASTES, NCSR “Demokritos”, 15341 Athens, Greece; kanelospan@gmail.com (P.K.); mlymperis@hotmail.com (E.L.); katerinakaloudi@yahoo.gr (A.K.); nock_berthold.a@hotmail.com (B.A.N.); 2 Molecular Pharmacology, School of Medicine, University of Crete, Heraklion, 70013 Crete, Greece; 3 Department of Radiology & Nuclear Medicine Erasmus MC, 3015 CN Rotterdam, The Netherlands; m.hendriks-dejong@erasmusmc.nl; 4 Cyclotron Rotterdam BV, Erasmus MC, 3015 CE Rotterdam, The Netherlands; erickrenning@gmail.com

**Keywords:** gastrin-releasing peptide receptor targeting, [^99m^Tc]Tc-radiotracer, tumor targeting, [^99m^Tc]Tc-DB1 mimic, PEG_x_-spacer, neprilysin-inhibition, phosphoramidon

## Abstract

*Background*: The frequent overexpression of gastrin-releasing peptide receptors (GRPRs) in human cancers provides the rationale for delivering clinically useful radionuclides to tumor sites using peptide carriers. Radiolabeled GRPR antagonists, besides being safer for human use, have often shown higher tumor uptake and faster background clearance than agonists. We herein compared the biological profiles of the GRPR-antagonist-based radiotracers [^99m^Tc]Tc-[N_4_-PEGx-DPhe^6^,Leu-NHEt^13^]BBN(6-13) (N_4_: 6-(carboxy)-1,4,8,11-tetraazaundecane; PEG: polyethyleneglycol): (i) [^99m^Tc]Tc-DB7 (x = 2), (ii) [^99m^Tc]Tc-DB13 (x = 3), and (iii) [^99m^Tc]Tc-DB14 (x = 4), in GRPR-positive cells and animal models. The impact of in situ neprilysin (NEP)-inhibition on in vivo stability and tumor uptake was also assessed by treatment of mice with phosphoramidon (PA). *Methods*: The GRPR affinity of DB7/DB13/DB14 was determined in PC-3 cell membranes, and cell binding of the respective [^99m^Tc]Tc-radioligands was assessed in PC-3 cells. Each of [^99m^Tc]Tc-DB7, [^99m^Tc]Tc-DB13, and [^99m^Tc]Tc-DB14 was injected into mice without or with PA coinjection and 5 min blood samples were analyzed by HPLC. Biodistribution was conducted at 4 h postinjection (pi) in severe combined immunodeficiency disease (SCID) mice bearing PC-3 xenografts without or with PA coinjection. *Results*: DB7, -13, and -14 displayed single-digit nanomolar affinities for GRPR. The uptake rates of [^99m^Tc]Tc-DB7, [^99m^Tc]Tc-DB13, and [^99m^Tc]Tc-DB14 in PC-3 cells was comparable and consistent with a radioantagonist profile. The radiotracers were found to be ≈70% intact in mouse blood and >94% intact after coinjection of PA. Treatment of mice with PA enhanced tumor uptake. *Conclusions*: The present study showed that increase of PEG-spacer length in the [^99m^Tc]Tc-DB7–[^99m^Tc]Tc-DB13–[^99m^Tc]Tc-DB14 series had little effect on GRPR affinity, specific uptake in PC-3 cells, in vivo stability, or tumor uptake. A significant change in in vivo stability and tumor uptake was observed only after treatment of mice with PA, without compromising the favorably low background radioactivity levels.

## 1. Introduction

The gastrin-releasing peptide receptor (GRPR) has attracted much attention in nuclear oncology owing to its high-density expression in frequently occurring human cancers, such as prostate cancer, mammary carcinoma, and others [1,2,3,4,5,6,7]. This finding can be elegantly exploited to direct diagnostic and therapeutic radionuclides to tumor sites by means of suitably designed peptide carriers that specifically interact with the GRPR on tumor cells [8,9,10,11]. Diagnostic imaging with gamma emitters (e.g., ^99m^Tc, ^111^In) for single-photon-emission computed tomography (SPECT) or positron emitters (e.g., ^68^Ga, ^64^Cu) for positron-emission tomography (PET) will allow for initial diagnosis, assessment of disease spread and progression, and selection of patients eligible for subsequent radionuclide therapy. Molecular imaging is likewise essential for dosimetry, therapy planning, and follow-up, enabling a patient-tailored, “theranostic” approach. Therapy per se is conducted with the respective peptide analog carrying a suitable particle emitter (beta, alpha, or Auger electron emitter).

Several analogs of the frog 14 peptide bombesin (BBN; Pyr–Gln–Arg–Leu–Gly–Asn–Gln–Trp–Ala–Val–Gly–His–Leu–Met–NH_2_) and its C-terminal BBN(6–14) fragment, showing high GRPR affinity, have been derivatized with the appropriate chelator for stable binding of the selected medically relevant radiometal and have been evaluated in animal models and in humans [8,11]. It should be noted that such analogs internalize into target cells and display agonistic profiles at the GRPR. Agonism at the GRPR, however, translates into adverse effects elicited in patients after intravenous injection of BBN analogs and the GRPR activation that follows [12,13,14]. Such effects intensify at the higher peptide doses administered during radionuclide therapy, thereby restricting the broader clinical use of GRPR agonists. A subsequent shift of paradigm from GRPR radioagonists to antagonists revealed unexpected benefits in their use beyond the anticipated inherent biosafety. The clearance GRPR radioantagonists, in contrast to agonists, turned out to be much faster from physiological tissues than from tumor sites [15]. The basis for this clinically appealing feature has not been elucidated yet, although it has been observed for other receptor radioantagonists as well [16].

**Figure 1:**
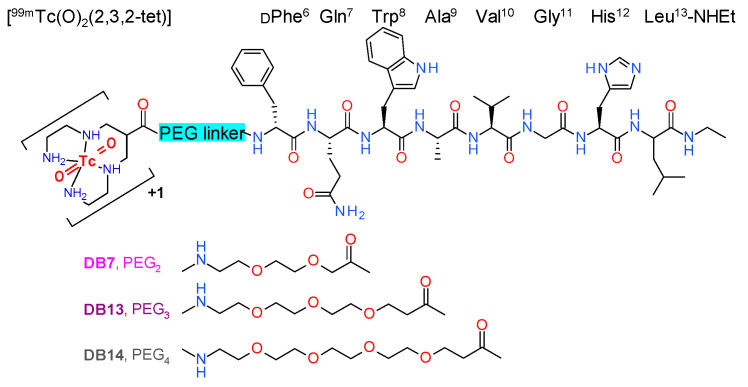
Chemical structure of [^99m^Tc]Tc-DB7 (PEG_2_), [^99m^Tc]Tc-DB13 (PEG_3_), and [^99m^Tc]Tc-DB14 (PEG_4_).

We previously reported on a series of radiolabeled analogs of the potent GRPR antagonist [H-DPhe^6^,Leu-NHEt^13^]BBN(6–13) [17,18], generated by coupling suitable chelators to the N-terminus via different linkers, which showed attractive pharmacokinetic profiles [19,20,21,22,23]. Thus, 1,4,8,11-tetraazaundecane has been used for labeling with the pre-eminent SPECT radionuclide [^99m^Tc]Tc, forming octahedral monocationic *trans*-dioxo Tc-complexes [24]. The resulting radiotracers, [^99m^Tc]Tc-DB1 mimics, have displayed high GRPR affinity, fair metabolic stability in peripheral mouse blood, and rapid localization in experimental xenografts in mice, whereas background clearance rates varied. [^99m^Tc]Tc-DB7, whereby 6-(carboxy)-1,4,8,11-tetraazaundecane (N_4_) is coupled to the peptide N-terminus via a polyethyleneglycol (PEG)_2_ spacer (Figure 1), showed the highest in vivo metabolic stability and tumor-to-pancreas ratio in mouse models [23].

In the present study, we designed two further [^99m^Tc]Tc-DB1 mimics, with the N_4_ coupled to the peptide chain via PEG_x_ linkers of increasing chain-length: [^99m^Tc]Tc-DB13 (x = 3), and [^99m^Tc]Tc-DB14 (x = 4) (Figure 1). We were interested to investigate the effect of linker length on several biological features of resulting analogs, such as GRPR affinity, cell uptake, in vivo metabolic stability, and pharmacokinetics in mice bearing human GRPR-expressing prostate adenocarcinoma PC-3 xenografts. A further objective of this study was to assess potential improvements of the PC-3 tumor targeting and overall pharmacokinetics of [^99m^Tc]Tc-DB7, [^99m^Tc]Tc-DB13, and [^99m^Tc]Tc-DB14 during transient inhibition of neprilysin (NEP) [25,26]. The latter was accomplished by coinjection of the NEP-inhibitor phosphoramidon (PA) [27] together with each radiotracer. This methodology was previously shown to enhance the metabolic stability of BBN and other peptide radioligands in peripheral blood and to improve the supply of the intact radiopeptide form to tumor sites. As a result, notably improved tumor targeting was observed in mice and recently also in patients [28,29,30,31,32,33,34,35].

## 2. Results

### 2.1. Radiolabeling and Quality Control

Radiolabeling of DB7, DB13, and DB14 with [^99m^Tc]Tc was accomplished by 30 min incubation at room temperature in alkaline aqueous medium containing citrate anions and SnCl_2_ as reductant. Quality control of the radiolabeled products (Figure 1) included HPLC and ITLC analysis and revealed less than 2% total radiochemical impurities ([^99m^Tc]TcO_4_^−^, [^99m^Tc]Tc-citrate, and [^99m^Tc]TcO_2_ × nH_2_O). A single radiopeptide species was obtained at molecular activities of 20–40 MBq [^99m^Tc]Tc/nmol peptide. In view of the above, [^99m^Tc]Tc-DB7, [^99m^Tc]Tc-DB13, and [^99m^Tc]Tc-DB14 were used without further purification in all subsequent assays.

### 2.2. In Vitro Assays in PC-3 Cells

#### 2.2.1. GRPR Affinity of Peptide Conjugates

**Figure 2:**
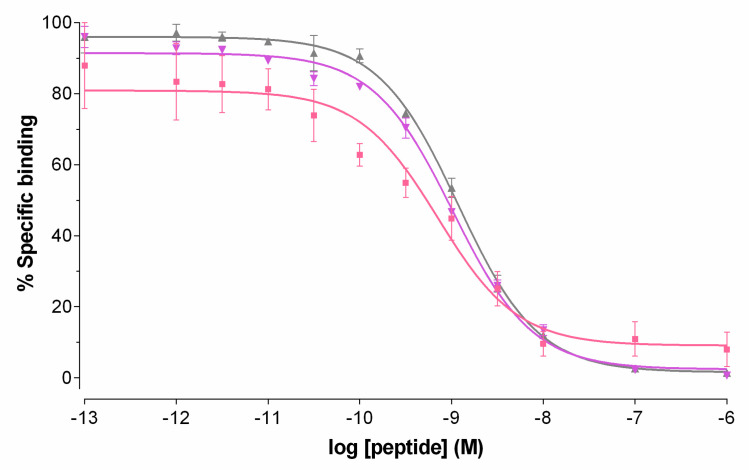
Displacement of [^125^I][I-Tyr^4^]BBN from GRPR binding sites in PC-3 cell membranes by increasing concentrations of DB7 (■, IC_50_ = 0.93 ± 0.01 nM), DB13 (▼, IC_50_ = 1.03 ± 0.01 nM), and DB14 (▲, IC_50_ = 1.18 ± 0.09 nM); results represent average values ± SD of three experiments performed in triplicate.

Competition binding assays for DB7, DB13 and DB14 were performed in PC-3 cell membranes. As shown in Figure 2, all three peptides were able to displace [^125^ I][I-Tyr^4^]BBN from GRPR binding sites on the membranes in a monophasic and dose-dependent manner. The binding affinities of the three analogs for the human GRPR were found comparable, DB7 (IC_50_ = 0.93 ± 0.01 nM), DB13 (IC_50_ = 1.03 ± 0.01 nM) and DB14 (IC_50_ = 1.18 ± 0.09 nM), indicating little influence of the PEG_x_-chain length.

#### 2.2.2. Radiotracer Uptake in PC-3 Cells

**Figure 3:**
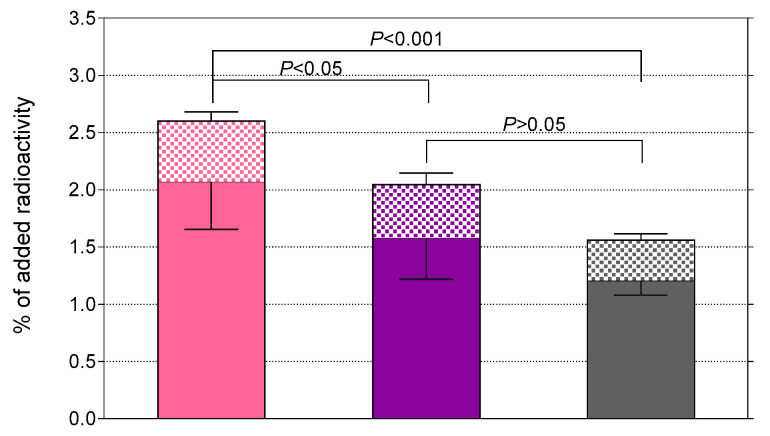
Specific uptake of [^99m^Tc]Tc-DB7 (pink bars), [^99m^Tc]Tc-DB13 (violet bars) and [^99m^Tc]Tc-DB14 (gray bars) in PC-3 cells after 1 h incubation at 37 °C (checkered bars: internalized, solid bars: membrane bound fractions); results represent the mean ± SD of 3 experiments performed in triplicate.

The uptake of [^99m^Tc]Tc-DB7, [^99m^Tc]Tc-DB13, and [^99m^Tc]Tc-DB14 in PC-3 cells is compared in Figure 3. In all cases, the bulk of radioactivity was found on the membrane of PC-3 cells with only a small portion detected within the cells, consistent with a noninternalizing radioantagonist profile [15,21]. Cell association was banned (<0.2%) in the presence of 1 μM [Tyr^4^]BBN, suggesting a GRPR-mediated process (results not shown). A decline in cell uptake was observed with increasing PEG-chain length. Thus, [^99m^Tc]Tc-DB7 (PEG_2_, 2.6 ± 0.5%) showed superior cell uptake compared with [^99m^Tc]Tc-DB13 (PEG_3_, 2.0 ± 0.5%; *p* < 0.05) and [^99m^Tc]Tc-DB14 (PEG_4_, 1.6 ± 0.2%; *p* < 0.001).

### 2.3. In Vivo Comparison of [^99m^Tc]Tc-DB7, [^99m^Tc]Tc-DB13, and [^99m^Tc]Tc-DB14

#### 2.3.1. Metabolic Studies in Mice

**Figure 4:**
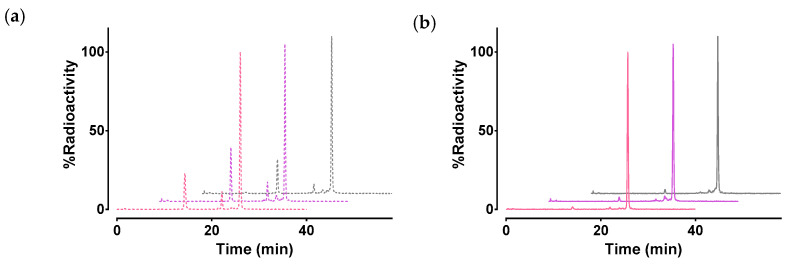
Representative HPLC radiochromatograms (System 2) of mouse blood collected 5 min after iv injection of [^99m^Tc]Tc-DB7 (pink lines), [^99m^Tc]Tc-DB13 (violet lines), and [^99m^Tc]Tc-DB14 (gray lines) (**a**) without (dotted lines) or (**b**) with PA coinjection (solid lines); results represent average values ± SD, *n* = 3.

The stability of [^99m^Tc]Tc-DB7, [^99m^Tc]Tc-DB13, and [^99m^Tc]Tc-DB14 in peripheral mouse blood was assessed at 5 min postinjection (pi) via HPLC analysis of blood samples. Representative radiochromatograms are shown in Figure 4a, revealing a 30% radiometabolite formation, and a comparable stability across radiotracers (≈70% intact radiopeptide, *p* > 0.05). After treatment of mice with PA, radiotracer stability was significantly enhanced (Figure 4b), namely, [^99m^Tc]Tc-DB7: 70.6 ± 1.1% to 94.5 ± 1.1% intact (*p* < 0.0001); [^99m^Tc]Tc-DB13: 71.2 ± 3.2% to 94.2 ± 1.3% intact (*p* < 0.0001); and [^99m^Tc]Tc-DB14: 77.9 ± 3.8% to 96.0 ± 1.0% intact (*p* < 0.001). These results implicate NEP in the partial in vivo degradation of the three radioligands [28,36,37].

#### 2.3.2. Biodistribution in PC-3 Tumor-Bearing Mice

Cumulative biodistribution data for [^99m^Tc]Tc-DB7, [^99m^Tc]Tc-DB13, and [^99m^Tc]Tc-DB14 in severe combined immunodeficiency disease (SCID) mice bearing PC-3 xenografts at 4 h pi, as %injected activity per gram tissue (%IA/g) ± SD, can be found in Table 1 ([^99m^Tc]Tc-DB7), Table 2 ([^99m^Tc]Tc-DB13), and Table 3 ([^99m^Tc]Tc-DB14).

All three radiotracers showed a fast blood and body clearance via the kidneys into the urine, displaying low background radioactivity uptake even in the GRPR-rich pancreas [38] (<1%IA/g pancreas for all three tracers). Uptake in the experimental PC-3 tumor was comparable across compounds in control mice, [^99m^Tc]Tc-DB7: 4.49 ± 1.20%IA/g, [^99m^Tc]Tc-DB13: 4.14 ± 0.78%IA/g, and [^99m^Tc]Tc-DB14 3.71 ± 1.04%IA/g (*p* > 0.05). This uptake was reduced in the block animal groups for [^99m^Tc]Tc-DB7 (*p* < 0.0001), [^99m^Tc]Tc-DB13 (*p* < 0.0001), and [^99m^Tc]Tc-DB14 (*p* < 0.01), indicating GRPR-specificity.

**Figure 5:**
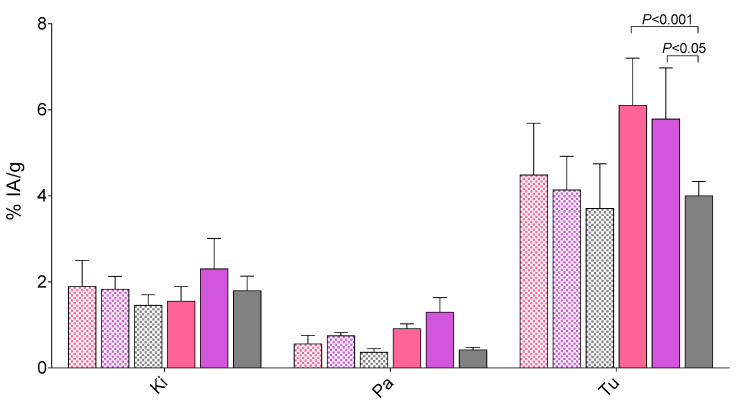
Selected biodistribution data for [^99m^Tc]Tc-DB7 (pink bars), [^99m^Tc]Tc-DB13 (violet bars), and [^99m^Tc]Tc-DB14 (gray bars) for kidneys (Ki), pancreas (Pa), and PC-3 tumors (Tu) at 4 h pi in female SCID mice bearing subcutaneous PC-3 xenografts; results are expressed as %IA/g mean ± SD, *n* = 4, without (checkered bars) or with coinjection (solid bars) of PA. Only statistical differences in organ/tumor uptake across compounds are included in the diagram; statistical differences between PA-treated and nontreated mice are shown in Table 1, Table 2, and Table 3.

During NEP-inhibition the uptake of the three radiotracers increased in the PC-3 tumors, [^99m^Tc]Tc-DB7: 6.10 ± 1.20%IA/g (*p* < 0.0001); [^99m^Tc]Tc-DB13: 5.79 ± 1.18%IA/g (*p* < 0.001); [^99m^Tc]Tc-DB14: 4.00 ± 0.34%IA/g (*p* > 0.05). Interestingly, neither renal uptake nor pancreatic uptake showed any significant increase after treatment of mice with PA. Representative data for the three radiotracers in kidneys, pancreas and tumor are depicted in Figure 5.

## 3. Discussion

Radiotracers based on GRPR antagonists have lately attracted much attention in nuclear medicine, largely because of their higher inherent safety for human use compared with agonists [20]. After intravenous injection to patients, antagonists seek and bind but do not activate the GRPR, and hence they do not elicit adverse effects. Furthermore, GRPR radioantagonists often display attractive pharmacokinetic profiles in preclinical models and in patients, as a combined result of rapid clearance from physiological tissues and good retention in tumor sites. In fact, a general concern in the application of GRPR radioligands for cancer theranostics has been the high-density expression of GRPR not only on tumors, but also in physiological tissues, especially in the pancreas [38]. Previous studies with both GRPR radioagonists and antagonists have demonstrated the significant impact of linkers, introduced between the metal chelate and the peptide part, on pharmacokinetics [23,39,40,41,42].

In line with these observations, we also noted marked differences in the pharmacokinetic profiles, and especially in the tumor vs. pancreas uptake, across a series of [^99m^Tc]Tc-based GRPR radioantagonists, [^99m^Tc]Tc-DB1 mimics [23]. These carry an acyclic tetraamine chelator via different types and lengths of linkers at the N-terminus of the potent GRPR antagonist [H-DPhe^6^,Leu-NHEt^13^]BBN(6-13) [17,18]. The PEG_2_-derivatized member of this series, [^99m^Tc]Tc-DB7 (Figure 1), displayed superior tumor over pancreas uptake, as well as considerably better metabolic stability in peripheral mouse blood.

In the present study, we were interested to find out whether the elongation of the PEG_2_ chain would further improve the biological properties of resulting analogs. For this purpose, [^99m^Tc]Tc-DB13 (PEG_3_) and [^99m^Tc]Tc-DB14 (PEG_4_) were newly synthetized and compared to [^99m^Tc]Tc-DB7 (Figure 1). As revealed by competitive binding assays in PC-3 cell membranes, the elongation of PEG_2_, to PEG_3_ and PEG_4_ had no apparent impact on the GRPR affinity of the respective DB7, DB13, and DB14 (Figure 2). On the other hand, the resultant [^99m^Tc]Tc radiotracers showed modest, but statistically significant, decline of uptake in PC-3 cells in vitro as the length of the PEG linker increased from PEG_2_ to PEG_4_ (Figure 3).

In a subsequent step, we investigated the effect of PEG-chain length on the metabolic stability of the three [^99m^Tc]Tc radiotracers in mouse blood collected 5 min pi. This study did not reveal any statistically significant differences across radiotracers (Figure 4a). NEP has been shown to be a major protease in the rapid in vivo degradation of BBN and its analogs [28,36,37]. NEP is actually an ectoenzyme abundantly present on the epithelial cells of several tissues of the body, including vasculature walls, kidneys, lungs, and intestines, but found only in minute amounts in the blood solute [25,26]. Therefore, its action is overlooked during in vitro incubation assays of radioligands in plasma or serum. We previously demonstrated that coinjection of NEP inhibitors, such as PA [27], with BBN-like radiopeptides, both agonists and antagonists, improves their metabolic stability in circulation [28,30,31,32,33,34]. As a result, an appreciably higher amount of intact radiopeptide eventually reaches tumor sites. Accordingly, tumor uptake is markedly enhanced, with clear benefits to be gained both for imaging and therapy. Following this rationale, we decided to study the effects of in situ inhibition of NEP on the in vivo stability of [^99m^Tc]Tc-DB7, [^99m^Tc]Tc-DB13, and [^99m^Tc]Tc-DB14 coinjected with PA in the present work. It is interesting to note that once again, significant enhancement of metabolic stability was documented for all three analogs (Figure 4b).

In order to assess how the above properties translate in terms of pharmacokinetics, biodistribution profiles of [^99m^Tc]Tc-DB7, [^99m^Tc]Tc-DB13, and [^99m^Tc]Tc-DB14 were compared in mice bearing GRPR-positive experimental tumors at 4 h pi, without or with PA coinjection. Firstly, we observed declining, but not statistically significant lower, uptake in the PC-3 tumors with increasing length of the PEG chain from 2 to 4 (Figure 5), as a combined result of (i) the equivalent GRPR affinities of DB7, DB13, and DB14, (ii) the slightly declining PC-3 cell uptake capabilities of [^99m^Tc]Tc-DB7, [^99m^Tc]Tc-DB13, and [^99m^Tc]Tc-DB13, and (iii) their similar metabolic stability. Secondly, overall pharmacokinetics turned out to be very comparable for all three radioantagonists, characterized by favorably low background radioactivity levels. Of particular advantage are the low radioactivity values displayed by the three radioligands in the GRPR-rich pancreas, as well as in the kidneys. Thirdly, treatment of mice with PA led to significant increase of tumor uptake compared with controls (Table 1, Table 2, and Table 3) without provoking any unfavorable rise of background activity, thereby further enhancing tumor to background contrast. Interestingly, the enhanced tumor uptake induced by PA via in situ stabilization of analogs in mouse circulation ended up being reflected in statistically significant differences between the PEG_2/3_ and PEG_4_ members of the series (Figure 5).

In conclusion, the effect of PEG_X_ linker length (x = 2, 3 and 4) in a series of GRPR radioantagonists, ([^99m^Tc]Tc-[N_4_-PEGx-DPhe^6^,Leu-NHEt^13^]BBN(6-13), Figure 1) had little effect on GRPR affinity, binding in GRPR-positive PC-3 cells, metabolic stability in mouse circulation, or PC-3 tumor targeting and overall pharmacokinetics in animal models. In all cases, tumor-to-background levels were favorable, including those in the GRPR-rich pancreas and the kidneys. Similar observations have been made for other GRPR antagonists, wherein the influences of different length of PEG linkers (x = 2, 3, 4) and different chelating moieties (NOTA: 1,4,7-triazacyclononane-1,4,7-triacetic acid, NODAGA: 1,4,7-triazacyclononane-1-(glutaric acid)-4,7-diacetic acid, DOTA: 1,4,7,10-tetraazacyclododecane-1,4,7,10-tetraacetic acid, DOTAGA: 1,4,7,10-tetraazacyclododececane-1-(glutaric acid)-4,7,10-triacetic acid) were distinct [39,40,42]. The effects of the PEG spacer’s length on the in vivo pharmacokinetics of resulting radioligands were found to be minor compared with those of the metal chelate or the peptide chain applied. In all cases, however, the use of PEG linkers was shown to favor in vivo metabolic stability and to boost background clearance, especially from the pancreas. Notably, the above attractive properties of [^99m^Tc]Tc-DB7, [^99m^Tc]Tc-DB13, and [^99m^Tc]Tc-DB14 were further enhanced by in situ NEP inhibition, inducing tumor uptake increases without affecting the advantageously low background radioactivity levels.

## 4. Materials and Methods

### 4.1. Chemicals and Radionuclides

All chemicals were reagent-grade and were therefore used without further purification. The peptide conjugates DB7, DB13, and DB14 were synthesized on a solid support and obtained from PiChem (Graz, Austria). [Tyr^4^]BBN (Pyr–Gln–Arg–Tyr–Gly–Asn–Gln–Trp–Ala–Val–Gly–His–Leu–Met–NH_2_) was purchased from Bachem (Bubendorf, Switzerland). PA (phosphoramidon disodium dehydrate, N-(α-rhamnopyranosyloxyhydroxyphosphinyl)-L-leucyl-L-tryptophan × 2Na × 2H_2_O) was obtained from PeptaNova GmbH (Sandhausen, Germany).

Technetium-99m in the form of [^99m^Tc]NaTcO_4_ was collected by elution of a [^99^Mo]Mo/[^99m^Tc]Tc generator (Ultra-Technekow™ V4 Generator, Curium Pharma, Petten, The Netherlands), while [^125^I]NaI in a solution of 10^−5^ M NaOH (10 μL) was purchased from Perkin Elmer (Waltham, MA, USA).

#### 4.1.1. Radiolabeling

The lyophilized peptide analogs were dissolved in water to a final concentration of 1 mM and 50 μL aliquots were stored at −20 °C. Labeling with [^99m^Tc]Tc was performed in an Eppendorf vial containing 0.5 M phosphate buffer (pH 11.5, 50 µL). [^99m^Tc]NaTcO_4_ eluate (410 µL, 370-550 MBq) was added to the vial followed by 0.1 M sodium citrate (5 µL), the peptide stock solution (15 µL, 15 nmol), and a freshly prepared SnCl_2_ solution in ethanol (20 µL, 20 μg). The mixture was left to react for 30 min at room temperature and the pH was neutralised with the addition of 0.1 M HCl.

Radioiodination of [Tyr^4^]BBN was performed following the chloramine-T method and any sulfoxide (Met^14^=O) that formed was reduced back to nonoxidized Met^14^ by dithiothreitol. The [^125^I-Tyr^4^]BBN was isolated in a highly pure form by HPLC and Met was added to the purified radioligand solution to prevent reoxidation of Met^14^ during storage; the resulting stock solution in 0.1% BSA-PBS was kept at −20 °C and aliquots thereof were used in competitive binding assays (74 GBq/μmol).

#### 4.1.2. Quality Control

Quality control comprised radioanalytical high-performance liquid chromatography (HPLC) and instant thin-layer chromatography (ITLC). HPLC analyses were performed on a Waters chromatograph coupled to a 996 photodiode array UV detector (Waters, Vienna, Austria) and a Gabi gamma detector (Raytest RSM Analytische Instrumente GmbH, Straubenhardt, Germany). Data processing and chromatography were controlled with the Empower Software (Waters, Milford, MA, USA). For analyses, a Symmetry Shield RP-18 (5 μm, 4.6 mm × 150 mm) cartridge column (Waters, Eschborn, Germany) was eluted at a 1 mL/min flow rate with a linear gradient system 1 starting from 0% B and advancing to 40% B within 20 min (solvent A = 0.1% aqueous TFA and B = MeCN). ITLC analyses were performed on Whatman 3 mm chromatography paper strips (GE Healthcare, Chicago, IL, USA), developed up to 10 cm from the origin with 5 M ammonium acetate/MeOH 1/1 (*v*/*v*) for the detection of reduced hydrolyzed technetium ([^99m^Tc]TcO_2_ × nH_2_O), or acetone for the detection of [^99m^Tc]TcO_4_^−^.

All manipulations with beta- and gamma-emitting radionuclides and their solutions were performed by trained and authorized personnel behind suitable shielding in licensed laboratories, in compliance with European radiation safety guidelines and supervised by the Greek Atomic Energy Commission (license #A/435/17092/2019)

### 4.2. In Vitro Assays

#### 4.2.1. Cell Lines and Culture

The human prostate adenocarcinoma PC-3 cell line spontaneously expressing GRPR [43] was obtained from LGC Standards GmbH (Wesel, Germany). All culture reagents were obtained from Gibco BRL, Life Technologies (Grand Island, NY, USA) or from Biochrom KG Seromed (Berlin, Germany). Cells were grown in Roswell Park Memorial Institute-1640 (RPMI-1640) medium with GlutaMAX-I supplemented with 10% (*v*/*v*) fetal bovine serum (FBS), 100 U/mL penicillin, and 100 μg/mL streptomycin, and kept in a controlled humidified air containing 5% CO_2_ at 37 °C. Splitting of cells with a ratio of 1:3 to 1:5 was performed as they approached confluency, using a trypsin/EDTA (0.05%/0.02% *w*/*v*) solution.

#### 4.2.2. Competitive Binding in PC-3 Cell Membranes

Competition binding experiments of DB7, DB14 and DB14 against [^125^ I][I-Tyr^4^]BBN were conducted in PC-3 cell membranes. Increasing concentrations of tested peptide (10^−13^–10^−5^ M) were mixed with the radioligand (40,000 cpm per assay tube, at a 50 pM concentration) and the membrane homogenate in a total volume of 300 μL binding buffer (pH 7.4, 50 mM HEPES, 1% BSA, 5.5 mM MgCl_2_, 35 μM bacitracin). Triplicates of each concentration point were incubated for 60 min at 22 °C in an Incubator-Orbital Shaker unit (MPM Instr. SrI, Bernareggio, MI, Italy). The incubation was interrupted by adding ice-cold washing buffer (10 mM HEPES pH 7.4, 150 mM NaCl), followed by rapid filtration over glass fiber filters (Whatman GF/B, presoaked in binding buffer) on a Brandel Cell Harvester (Adi Hassel Ingenieur Büro, Munich, Germany). Filters were washed with cold washing buffer and were counted for their radioactivity content in an automated well-type gamma counter [NaI(Tl) 3″ crystal, Canberra Packard Cobra Quantum series instrument]. The 50% inhibitory concentration (IC_50_) values were calculated by nonlinear regression according to a one-site model applying the PRISM 6 program (Graph Pad Software, San Diego, CA, USA) and are expressed as mean ± SD of three experiments performed in triplicate.

#### 4.2.3. Internalization of [^99m^Tc]Tc Radiotracers in PC-3 Cells

For internalization assays with [^99m^Tc]Tc-DB7, [^99m^Tc]Tc-DB13, and [^99m^Tc]Tc-DB14, PC-3 cells were seeded in six well plates (≈1 × 10^6^ cells per well) 24 h before the experiment. Cells were rinsed twice with ice-cold internalization medium (RPMI-1640 GlutaMAX-I, supplemented by 1% (*v*/*v*) FBS) and then fresh medium was added (1.2 mL) at 37 °C, followed by test radiopeptide (250 fmol total peptide in 150 μL 0.5% BSA-PBS, 100,000–200,000 cpm). Nonspecific internalization was determined by a parallel triplicate series containing 1 μM [Tyr^4^]BBN. After 1 h incubation at 37 °C, the plates were placed on ice, the medium was collected, and the plates were washed with 0.5% BSA-PBS (1 mL). Membrane-bound fractions were collected by incubating the cells 2 × 5 min in acid-wash solution (2 × 600 µL; 50 mM glycine buffer pH 2.8, 0.1 M NaCl) at room temperature. After rinsing the cells with 0.5% BSA-PBS (1 mL), internalized fractions were collected by lysing the cells with 1 N NaOH (2 × 600 µL). Sample radioactivity was measured on the gamma counter and the percentage, of specific internalized and membrane-bound fractions were calculated with Microsoft Excel (after subtracting nonspecific from overall internalized and membrane-bound counts). Results represent specific internalized ± SD of total added radioactivity per well from three experiments performed in triplicate.

### 4.3. Animal Studies

#### 4.3.1. Metabolic Studies in Mice

A bolus containing each of [^99m^Tc]Tc-DB7, [^99m^Tc]Tc-DB13, and [^99m^Tc]Tc-DB14 (100 μL, 55.5–111 MBq, 3 nmol of total peptide in vehicle: saline/EtOH 9/1 *v*/*v*) was injected into the tail vein of healthy male Swiss albino mice, together with vehicle (100 μL; control group), or PA (100 μL of vehicle containing 300 μg PA; PA group). Animals were euthanized 5 min pi and blood was collected and immediately placed in prechilled polypropylene vials containing EDTA on ice. Samples were centrifuged at 2000× *g* at 4 °C for 10 min, and the plasma was collected and mixed with an equal volume of MeCN and centrifuged again for 10 min at 15,000× *g* at 4 °C. The supernatant was collected and concentrated to a small volume under a gentle N_2_ flux at 40 °C, diluted with physiological saline (≈400 μL), and filtered through a Millex GV filter (0.22 μm). Suitable aliquots of the filtrate were analyzed by RP-HPLC on a Symmetry Shield RP18 (5 μm, 3.9 mm × 20 mm) column (Waters, Germany), eluted at a flow rate of 1 mL/min and adopting gradient system 2: 100% A/0% B to 50% A/50% B in 50 min; A = 0.1% TFA in H_2_O and B = MeCN (system 2). The elution time (*t*_R_) of intact radioligand was determined by coinjection with a sample of the labeling reaction solution.

#### 4.3.2. Biodistribution in SCID Mice Bearing PC-3 Xenografts

Suspensions of freshly harvested PC-3 cells (~150 μL, 1.4 × 10^7^) in normal saline was subcutaneously inoculated into the flanks of 6 week old SCID mice (NCSR “Demokritos” Animal House, 16.01 ± 2.59 g body weight). After 3–4 weeks, palpable PC-3 tumors (108.49 ± 0.04 mg) had developed at the inoculation sites and biodistribution was performed. At the day of the experiment, a bolus of [^99m^Tc]Tc-DB7, [^99m^Tc]Tc-DB13, or [^99m^Tc]Tc-DB14 (180-230 kBq, 10 pmol total peptide, in vehicle: saline/EtOH 9/1 *v*/*v*) was intravenously injected into the tail of each mouse together with either vehicle (100 μL; control group), PA (300 μg PA dissolved in 100 μL vehicle; PA group), or excess [Tyr^4^]BBN (50 μg [Tyr^4^]BBN dissolved in 100 μL vehicle; block group). Animals were euthanized at 4 h pi in groups of four and blood samples, organs of interest, and tumors were dissected, weighed, and counted in the gamma counter. Biodistribution data were calculated as percent of injected activity per gram of tissue (%IA/g) with the aid of suitable standards of the injected dose, using the Microsoft Excel program. Results represent average values ± SD, *n* = 4.

#### 4.3.3. Statistical Analysis

For statistical analysis of biological results, a two-way ANOVA with multiple comparisons was used applying Tukey’s post hoc analysis (GraphPad Prism Software, San Diego, CA, USA). *p*-values of <0.05 were considered to be statistically significant.

All animal studies were performed in compliance with European guidelines in supervised and licensed facilities (EL 25 BIO 021), and the study protocols were approved by the Department of Agriculture and Veterinary Service of the Prefecture of Athens (protocol numbers #1609 for the stability studies and #1610 for biodistribution and imaging studies)

## Figures and Tables

**Table 1:** Biodistribution data for [^99m^Tc]Tc-DB7, expressed as %injected activity per gram tissue (%IA/g) mean ± SD, *n* = 4, in PC-3 xenograft-bearing severe combined immunodeficiency disease (SCID) mice at 4 h pi.

Tissue			[^99m^Tc]Tc-DB7		
	Block ^1^		Controls		PA ^2^
Blood	0.08 ± 0.03		0.05 ± 0.01		0.06 ± 0.02
Liver	2.11 ± 0.43		0.96 ± 0.23		0.87 ± 0.12
Heart	0.09 ± 0.03		0.04 ± 0.01		0.07 ± 0.03
Kidneys	2.38 ± 0.56		1.89 ± 0.61		1.55 ± 0.34
Stomach	0.45 ± 0.34		0.27 ± 0.17		0.22 ± 0.05
Intestines	2.85 ± 0.39		1.38 ± 0.27		1.64 ± 0.72
Spleen	1.33 ± 0.37		0.24 ± 0.07		0.28 ± 0.1
Muscle	0.02 ± 0.01		0.02 ± 0.01		0.02 ± 0.01
Lungs	0.55 ± 0.26		0.16 ± 0.12		0.14 ± 0.02
Pancreas	0.37 ± 0.09	⇤ *p* > 0.05 ⇥	0.56 ± 0.2	⇤ *p* > 0.05 ⇥	0.91 ± 0.12
Tumor	0.53 ± 0.20	⇤ *p* < 0.0001 ⇥	4.49 ± 1.20	⇤ *p* < 0.0001 ⇥	6.10 ± 1.1

All animals were injected with 180–230 kBq/10 pmol peptide; ^1^ animals co-injected with 50 µg [Tyr^4^]BBN for in vivo GRPR-blockade; ^2^ animals co-injected with 300 µg PA for in situ inhibition of NEP.

**Table 2:** Biodistribution data for [^99m^Tc]Tc-DB13, expressed as %IA/g mean ± SD, *n* = 4, in PC-3 xenograft-bearing SCID mice at 4 h pi.

Tissue			[^99m^Tc]Tc-DB13		
	Block ^1^		Controls		PA ^2^
Blood	0.09 ± 0.01		0.06 ± 0.02		0.19 ± 0.19
Liver	1.8 ± 0.15		0.75 ± 0.24		0.88 ± 0.25
Heart	0.18 ± 0.07		0.07 ± 0.03		0.16 ± 0.08
Kidneys	3.5 ± 1.59		1.83 ± 0.3		2.66 ± 1.14
Stomach	1.25 ± 0.86		0.9 ± 0.18		1.09 ± 0.47
Intestines	5.37 ± 1.34		3.69 ± 0.72		4.23 ± 0.98
Spleen	1.14 ± 0.47		0.29 ± 0.07		0.36 ± 0.13
Muscle	0.04 ± 0.02		0.03 ± 0.01		0.16 ± 0.32
Lungs	0.67 ± 0.31		0.13 ± 0.01		0.24 ± 0.08
Pancreas	0.3 ± 0.09	⇤ *p* > 0.05 ⇥	0.75 ± 0.08	⇤ *p* > 0.05 ⇥	1.82 ± 1.04
Tumor	1.12 ± 0.23	⇤ *p* < 0.0001 ⇥	4.14 ± 0.78	⇤ *p* < 0.001 ⇥	5.79 ± 1.18

All animals were injected with 180–230 kBq/10 pmol peptide; ^1^ animals co-injected with 50 µg [Tyr^4^]BBN for in vivo GRPR-blockade; ^2^ animals co-injected with 300 µg PA for in situ inhibition of NEP.

**Table 3:** Biodistribution data for [^99m^Tc]Tc-DB14, expressed as %IA/g mean ± SD, *n* = 4, in PC-3 xenograft-bearing SCID mice at 4 h pi.

Tissue			[^99m^Tc]Tc-DB14		
	Block ^1^		Controls		PA ^2^
Blood	0.21 ± 0.13		0.06 ± 0.03		0.07 ± 0.01
Liver	1.69 ± 0.15		0.79 ± 0.32		0.93 ± 0.13
Heart	0.23 ± 0.08		0.08 ± 0.05		0.15 ± 0.03
Kidneys	7.05 ± 4.24		1.46 ± 0.25		1.79 ± 0.34
Stomach	0.85 ± 0.10		0.75 ± 0.72		0.21 ± 0.06
Intestines	6.25 ± 2.51		2.03 ± 0.81		1.83 ± 0.77
Spleen	0.89 ± 0.15		0.19 ± 0.07		0.24 ± 0.05
Muscle	0.07 ± 0.04		0.04 ± 0.03		0.03 ± 0.01
Lungs	0.78 ± 0.25		0.15 ± 0.06		0.16 ± 0.05
Pancreas	0.32 ± 0.09	⇤ *p* > 0.05 ⇥	0.36 ± 0.09	⇤ *p* > 0.05 ⇥	0.42 ± 0.06
Tumor	2.16 ± 0.59	⇤ *p* < 0.01 ⇥	3.71 ± 1.04	⇤ *p* > 0.05 ⇥	4.00 ± 0.34

All animals were injected with 180–230 kBq/10 pmol peptide; ^1^ animals co-injected with 50 µg [Tyr^4^]BBN for in vivo GRPR-blockade; ^2^ animals co-injected with 300 µg PA for in situ inhibition of NEP
